# Teleostean fishes may have developed an efficient Na^+^ uptake for adaptation to the freshwater system

**DOI:** 10.3389/fphys.2022.947958

**Published:** 2022-10-05

**Authors:** Yung-Che Tseng, Jia-Jiun Yan, Fumiya Furukawa, Ruo-Dong Chen, Jay-Ron Lee, Yi-Ling Tsou, Tzu-Yen Liu, Yu-Hsin Tang, Pung-Pung Hwang

**Affiliations:** ^1^ Institute of Cellular and Organismic Biology, Academia Sinica, Taipei, Taiwan; ^2^ Kitasato University School of Marine Biosciences, Tokyo, Japan

**Keywords:** ammonotelic, teleosts, Na^+^-uptake, energy constraint, evolutionary selection

## Abstract

Understanding Na^+^ uptake mechanisms in vertebrates has been a research priority since vertebrate ancestors were thought to originate from hyperosmotic marine habitats to the hypoosmotic freshwater system. Given the evolutionary success of osmoregulator teleosts, these freshwater conquerors from the marine habitats are reasonably considered to develop the traits of absorbing Na^+^ from the Na^+^-poor circumstances for ionic homeostasis. However, in teleosts, the loss of epithelial Na^+^ channel (ENaC) has long been a mystery and an issue under debate in the evolution of vertebrates. In this study, we evaluate the idea that energetic efficiency in teleosts may have been improved by selection for ENaC loss and an evolved energy-saving alternative, the Na^+^/H^+^ exchangers (NHE3)-mediated Na^+^ uptake/NH_4_^+^ excretion machinery. The present study approaches this question from the lamprey, a pioneer invader of freshwater habitats, initially developed ENaC-mediated Na^+^ uptake driven by energy-consuming apical H^+^-ATPase (VHA) in the gills, similar to amphibian skin and external gills. Later, teleosts may have intensified ammonotelism to generate larger NH_4_^+^ outward gradients that facilitate NHE3-mediated Na^+^ uptake against an unfavorable Na^+^ gradient in freshwater without consuming additional ATP. Therefore, this study provides a fresh starting point for expanding our understanding of vertebrate ion regulation and environmental adaptation within the framework of the energy constraint concept.

## Introduction

The primitive evolution of vertebrates took place exclusively in the marine environments (Bartels and Potter, 2004), with early-branching vertebrates being both ionoconformers and osmoconformers (Willmer et al., 2009). In the marine seawater (SW) circumstance, living organisms such as teleosts would passively lose water and gain dissolved sodium (Na^+^) and chloride (Cl^−^) ions. For physiological compensation, they drink SW and actively excrete most monovalent ions majorly through the gills (Evans et al., 2005; Hwang and Lin, 2013). Those ionocyes in gill epithelia are the primary sites responsible for the active ion transport functions conducted by various ion transporters and enzymes (Hwang et al., 2011). Some vertebrates further invaded and colonized in freshwater (FW) or diluted-media ecosystems where the species would face problems of passive and continuous loss of Na^+^ and Cl^−^ ions. Due to a lower ionic strength than in marine environments, lower buffer capacity and more variable pH values are reasonably expected in the FW system (Tseng et al., 2020). To cope with the transition from SW to FW habitats, those FW conquerors may therefore be considered as pioneers of absorbing monovalent Na^+^ and Cl^−^ ions from FW environments. The colonization of FW habitats was thought as one of the most important advances in the evolution of vertebrate physiology (Halstead and Lawson, 1985). Maintaining homeostasis in the body fluids by developing iono/osmo-regulation mechanisms similar to those found in terrestrial mammals is also proved to be achieved and maintained by active uptake machineries *via* gill/skin (Hwang et al., 2011; Tseng et al., 2020). Besides, epithelial driving forced transports are high energy-consuming processes (Evans et al., 2005; Tseng et al., 2007; Tseng and Hwang, 2008; Hwang et al., 2011; Huang et al., 2020); the effects of environmental ionic perturbations on the metabolic provision in aquatic organisms have long fascinated researchers with energy constraint concerns (Huang et al., 2020). However, limited information is available to better elaborate on the ionic homeostasis physiology and metabolic adjustments underlying the evolutionary point of view.

**FIGURE 1:**
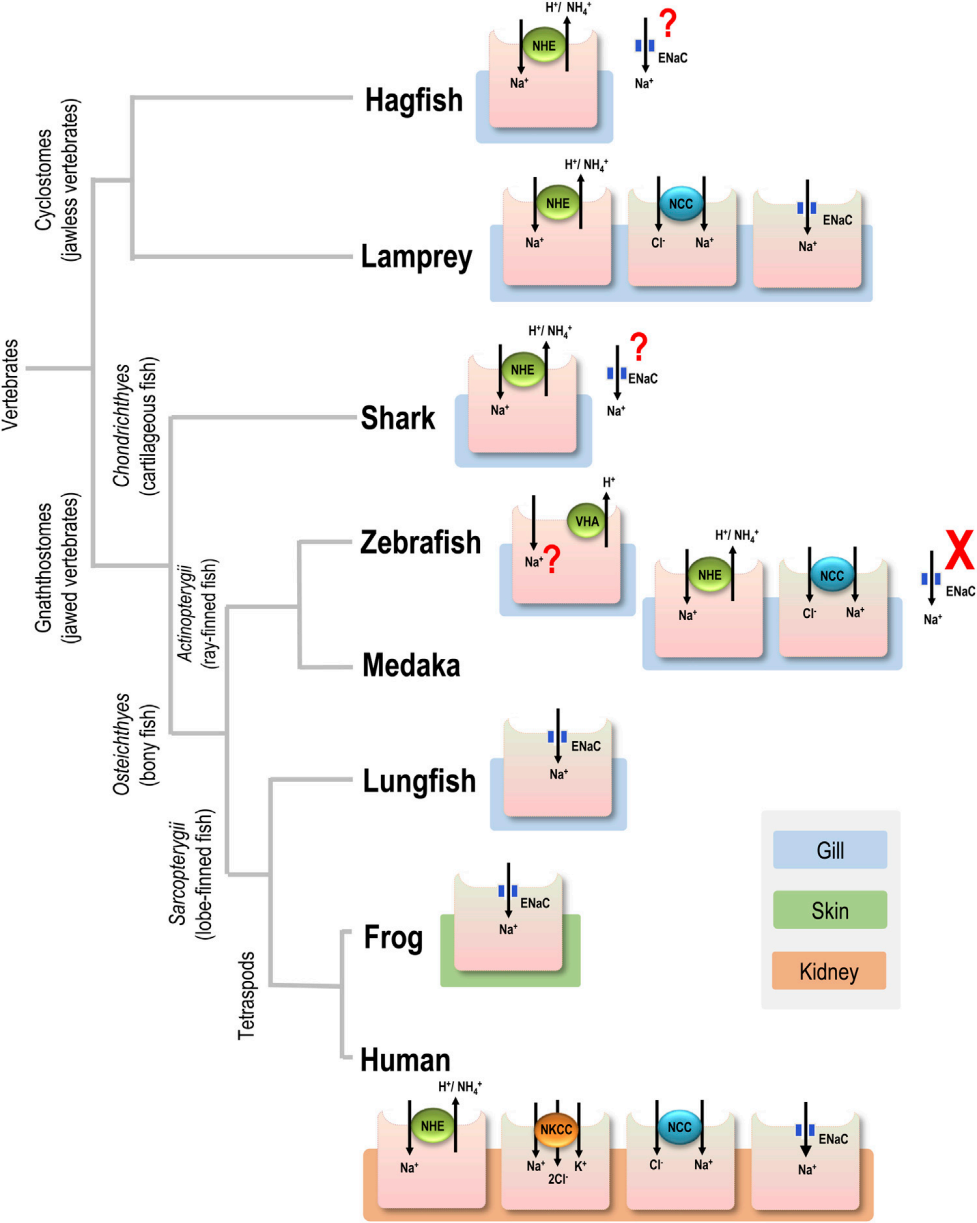
Major apical ion transporters for Na^+^ uptake in various vertebrates. For simplicity, only transporters in the gills of aquatic vertebrates, the skin of frog, and the kidney of human are shown. Ionocytes are responsible for Na^+^ uptake function. ENaC, epithelial Na^+^ channel; NCC, Na^+^-Cl^-^ cotransporter; NHE, Na^+^/H^+^ exchanger; NKCC, Na^+^-K^+^-2Cl^-^ cotransporter; question mark (?), transport pathway without physiological or cell biological characterization; cross mark (X), loss of the gene.

Na^+^ regulation in body fluids is essential for maintaining blood pressure and related physiological processes, in addition to modulating disease pathogenesis (Blaustein et al., 2012; Kirabo, 2017). In humans, the transport mechanisms of Na^+^ reabsorption in renal cells are the most important components of this regulatory process (Dantzler, 2003). However, it remains largely unknown how Na^+^ uptake mechanisms were developed and adapted during the evolution of vertebrates (Tseng et al., 2020; Barany et al., 2021). Compared to marine environments, FW habitats typically have low osmolarities (<1 mOsm), and Na^+^ concentrations (∼0.39 mM) that are less than those in body fluid (333 mOsm, 156 mM), so active Na^+^ absorption is required for body fluid osmotic and ionic homeostasis (Hwang and Lin, 2013). In mammalian kidneys, the Na^+^/H^+^ exchanger (NHE), Na^+^-K^+^-2Cl^-^ cotransporter (NKCC), Na^+^-Cl^-^ cotransporter (NCC), and epithelial Na^+^ channel (ENaC) are the major apical transporters that absorb Na^+^ to maintain body fluid Na^+^ homeostasis (Palmer and Schnermann, 2015). Most of these transport pathways driven by the primary or secondary active transport machinery were also characterized in the gills and kidney of teleosts (Hwang and Perry, 2010; Dymowska et al., 2012). However, ENaC genes and ENaC-mediated Na^+^ uptake function are not found in teleosts (Figure 1) (Hwang and Perry, 2010; Hwang et al., 2011). Although there have been major advances in understanding how the evolutionary success of teleosts with the additional teleosts-specific whole-genome duplication (TWGD) (Jaillon et al., 2004), the direct involvement of TWGD in every variety of teleosts physiological significances by the enrolled epithelial channels/transporters has so far been overlooked (Lin et al., 2020; Duran et al., 2021). Since the Na^+^ regulation is an essential physiological event and fundamental driving force requisite of any transepithelial transport, homeostasis is impossible without it. The absence of ENaC in teleosts has long been a mystery in the evolution of vertebrates, and most of the studies so far have approached the issue were limited from a genomic point of view; however, it is still an issue of debate (Wichmann and Althaus, 2020).

This study aimed to explore this evolutionary mystery regarding how the FW conquerors from the marine habitats developed the traits of absorbing Na^+^ ion from the Na^+^-poor FW circumstances for body fluid osmotic and ionic homeostasis from a comparative physiological point of view. Stenohaline lamprey (*Lethenteron reissneri*), a pioneer species invading FW habitats, and Japanese mekada (*Oryzias latipes*), a euryhaline teleost, were used to characterize the expression and function of ENaC, NHE, and H^+^-ATPase (VHA), and the effects of low-Na^+^ environment on the expression and function of the related transporters. Na^+^ uptake mechanisms were also compared between fishes (present study) and other vertebrate species (published in the literature). The present study may provide physiological insights into our understanding of how vertebrates originated in the hyperosmotic marine environments could develop adaptive strategies for body fluid ionic homeostasis during the course of evolution when they invaded and conquered the FW habitats. The environmental-driven traits in teleosts may also benefit our understanding of potential associations between evolutionary frameworks and adaptive homeostasis to make projections of climate change effects on organisms in future aquatic environments.

## Materials and methods

### Experimental animals and acclimation experiments

**FIGURE 2:**
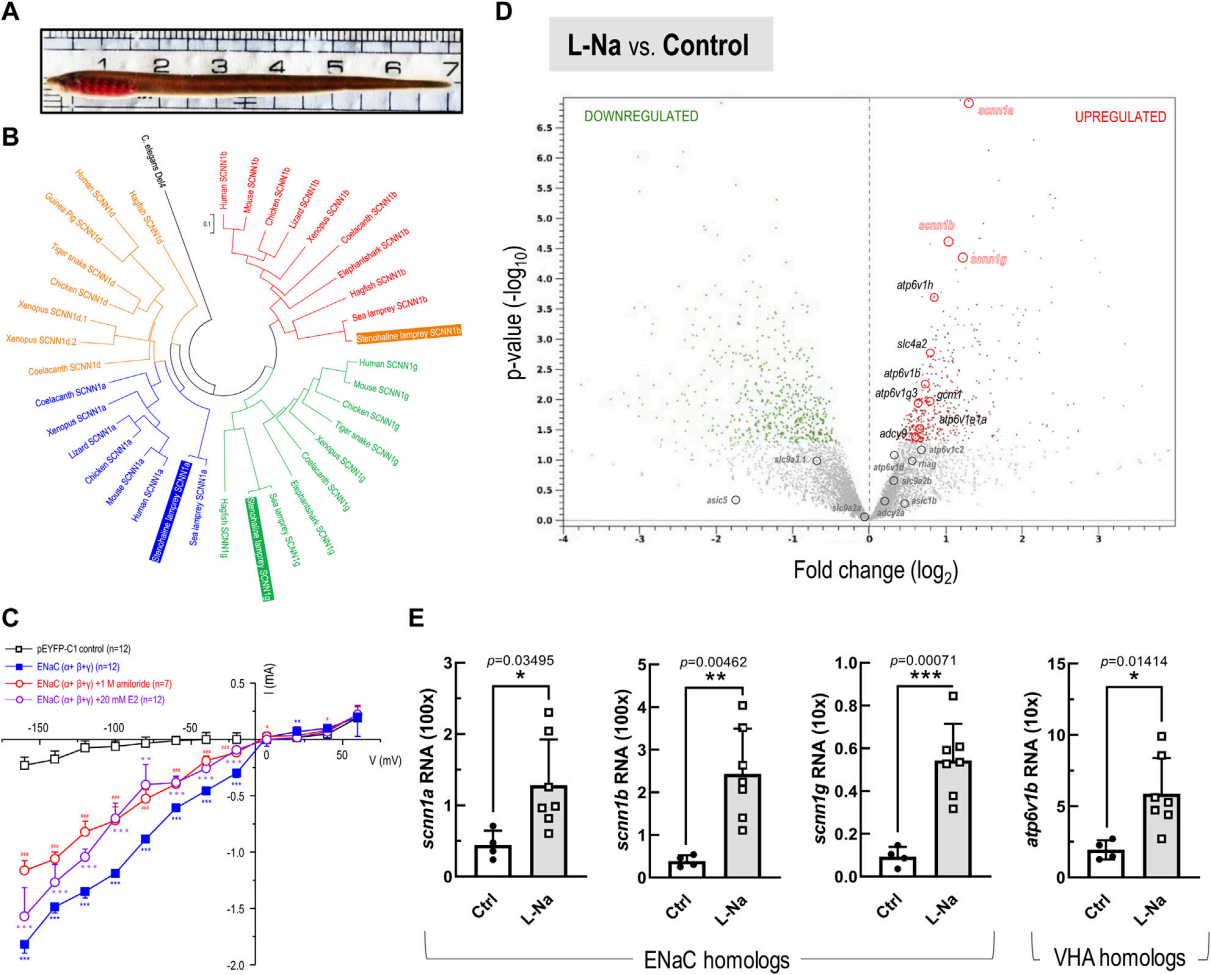
Phylogenetic with functional analysis of epithelial Na^+^ channel (ENaC), and RNA-Seq transcriptome profiling-based transcript expressions in the stenohaline lamprey (*Lethenteron reissneri*). (**A)**, Represented morphology of stenohaline lamprey applied in this study. **(B)**, Phylogenetic analysis of ENaC subtypes in various organisms. ENaC homologs could be sub-divided into four clusters, and both the stenohaline lamprey and the sea lamprey (*Petromyzon marinus*) ENaC consists of alpha (*scnn1a*), beta (*scnn1b*), and gamma (*scnn1g*) subunits. The *scnn1d* gene characterized in other vertebrates was lost in both the stenohaline- and sea-lampreys. The degenerin/epithelial Na^+^ channel in *Caenorhabditis elegans* was selected as the outgroup gene. **(C)**, Current-voltage relationships in the HEK293 cells expressing stenohaline lamprey ENaC (α+β+γ) and the control cells transfected the pEYFP-C1 plasmid. Data are expressed as mean ± SE. Asterisk (*), hashtag (#), and at-sign (∗) indicate significant differences between groups of ENaC(α+β+γ)-expressing cells, ENaC (α+β+γ)-expressing cells+1 M amiloride, and ENaC(α+β+γ)-expressing cells+20 mM estrogen (E2), respectively, compared with the group of expression vector (pEYP-C1)-injected cells (Student’s t-test). *p* < 0.05 (* or # or ∗∗), *p* < 0.01 (**or ## or ∗∗), and *p* < 0.001 (***or ### or ∗∗∗) indicate significant differences treatment conditions and control group. **(D)**, The volcano plot shows fold changes (represented as log_2_) *versus p*-values (represented as -log_10_) for the analyzed transcriptome profiling in the gills between low-Na^+^ (0.01 mM, L-Na) and control freshwater (1.05 mM, Ctrl) groups (*n* = 4 in each group). Based on differential expression analysis, the red and green dots, respectively, indicate transcripts with significantly upregulated and downregulated expression (*p*-value < 0.05) in the stenohaline lamprey gill under low-Na^+^ conditions compared to the control freshwater. Meanwhile, grey dots mark the transcripts with *p*-value > 0.05. **(E)**, mRNA expression levels of *scnn1a* (ENaC α), *scnn1b* (ENaC β), *scnn1g* (ENaC γ), and *atp6v1b* (VHA) in the gills of the stenohaline lamprey estimated by quantitative real-time PCR. Values were normalized by ribosomal protein L18 (*rpl18*). Data are expressed as mean ± SE. Asterisk indicates significant difference of *p* < 0.05 (*), *p* < 0.01 (**), or *p* < 0.001 (***) between low-Na^+^ condition and the control FW group (Student’s *t*-test).

Sex-unmatured ammocoetes larvae of stenohaline lamprey (Figure 2A) were collected in Murakami (Niigata, Japan) during March and April from 2016–2021, and Japanese medaka were from the stock maintained at Academia Sinica. Before the experiments, lamprey and medaka were kept at 10°C and 28°C, respectively, in control FW. For acclimation experiments, animals were transferred to control or low-Na^+^ FW for 7 days. The control and low-Na^+^ FW stocks were prepared by adding adequate amounts of MgSO_4_ ⋅ 7H_2_O, CaSO_4_ ⋅ 2H_2_O, NaCl, KH_2_PO_4_, K_2_HPO_4_, and CaCl_2_ to double distilled water, as described in Supplementary Table S1 (pH: 6.9–7.1). The medium was changed daily during the acclimation experiments to maintain water quality. No animals exhibited overtly abnormal behavior. To collect gill tissues, animals were euthanized with MS-222 (ethyl 3-aminobenzoate methanesulfonate salt, 0.03%) and dissected on ice. All experiments were conducted following protocols approved by the Academia Sinica Institutional Animal Care and Utilization Committee (approval no. BSF0414-00002941).

### RNA-seq transcriptome analysis

Gill tissue was collected from at least four individual stenohaline lampreys per group and prepared for total RNA extraction (QIAGEN RNeasy Plus Universal Mini Kit, Germany). The Illumina HiSeq 2000 platform (San Diego, CA, United States) with 150 bp paired-end sequencing libraries was used for RNA-seq, yielding 12 Gb data per sample. The RNAseq data were adequately trimmed and mapped to the sea lamprey (*Petromyzon marinu*s) genome database (Ensembl; Pmarinus_7.0) using the CLC genomics workbench (v. 20, CLC Bio-Qiagen, Aarhus, Denmark). The RNA-seq profiling was uploaded as the SRA data (Accession number: PRJNA794988) and then used to compile gene expression profiles as well as perform comparative analyses. Pairwise comparisons were performed on expression levels of each gene between treatments. Candidate transcripts were selected according to fold-change (represented as log_2_) and *p*-value (represented as -log_10_) for the transcriptome profiling analysis. Candidates were further validated by quantitative real-time quantitative PCR analysis.

### Gene cloning and quantification analysis

To test whether the identified *scnn1a*, *scnn1b*, and *scnn1g* candidates assembled from RNA-seq profiling encode ENaC orthologues, the deduced amino-acid sequences of the stenohaline lamprey genes were aligned with ClustalX together with known protein sequences from the genome database (Ensembl Genome Browser) or NCBI database (Supplementary Table S2). Phylogenetic analysis was applied using the maximum-likelihood (ML) method with 10,000-bootstrap replicate analyses using MEGA 5. Furthermore, specific primers (Supplementary Table S3) were designed in order to clone the candidate stenohaline lamprey genes by reverse-transcriptase polymerase chain reaction (RT-PCR).

For cDNA synthesis, 5 μg of mRNA was reverse transcribed in a final volume of 20 μl, containing 0.5 mM dNTPs, 2.5 mM oligo (dT)_20_, 250 ng random primers, 5 mM dithiothreitol, 40 units of RNase inhibitor, and 200 units of SuperScript III RT (Invitrogen, Carlsbad, CA, United States) for 1 h at 50°C, followed by incubation at 70°C for 15 min. The amount and quality of cDNA were determined at 260 and 280 nm by the Qubit dsDNA HS Assay Kit on the Qubit Fluorometer (Life Technologies, CA, United States). For PCR amplification, 1 μl of cDNA was used as a template in a 25 μl final reaction volume, containing 0.25 mM dNTPs, 2.5 units of Gen-Taq polymerase (GeneMark, Taipei, Taiwan), and 0.2 μM of each primer (Table S3). For each reaction, PCR was performed for 40 cycles. PCR products were then subcloned into a pGEM-T Easy vector (Promega, Madison, WI, United States of), and the nucleotide sequences were validated using an ABI 3730XL sequencer (Applied Biosystems, Warrington, United Kingdom). Sequence analysis, alignment, and confirmation were carefully conducted with both the BLASTx program (NCBI) and the BLAST/BLAT search program (Ensembl).

The mRNA expression levels of target genes were measured by quantitative real-time quantitative PCR analysis (qPCR) with a Roche LightCycler^®^ 480 System (Roche Applied Science, Mannheim, Germany). Each reaction had a total volume of 10 μl, including LightCycler^®^ 480 SYBR Green I Master (Roche) (5 μl), 10 μM forward and reverse primers (1 μl) (Table S3), and 1:100-diluted template cDNA (4 μl). All genes were tested using the following qPCR program: 1) pre-incubation: one cycle of 95°C for 5 min; 2) amplification: 45 cycles of 95°C for 10 s, 57/60°C for 10 s and 72°C for 10 s; 3) melting curve: 95°C for 5 s, 60°C for 1 min. The specificity of each primer set was confirmed by the presence of a single band with the expected size by gel electrophoresis. In addition, the melting-curve analysis showed a single peak for the products of each primer pair. A standard curve for each gene was used to find the linear range. Ribosomal protein L18 (*rpl18*) was used as an internal control. Control reactions were conducted with sterile water in place of the template to determine background signal levels.

### Expression and functional characterization of lamprey epithelial sodium channel subunits in HEK293 cells

The full-length open reading frame of lamprey ENaCα, ENaCβ, and ENaCγ were PCR amplified. The resulting amplicons of ENaCα and ENaCβ were digested with HindIII/KpnI, while the ENaCγ amplicon was digested with XhoI/SmaI, and subsequently ligated to the multiple cloning site of pEYFP-C1 plasmid (Clontech, Palo Alto, CA, United States) with an epitope tag eYFP at the N-terminus to generate the EYFP-ENaC fusion constructs. The human embryonic kidney 293 cell line (HEK293) was applied for lamprey ENaC electrophysiological whole-cell patch recordings while transfected with the DNA constructs. The HEK293 cells were cultured at a density of 200,000–400,000 cells/well in the 6-well plate and maintained in Dulbecco’s modified Eagle’s medium (Gibco, Grand Island, NY, United States) supplemented with 10% fetal bovine serum (VWR, Visalia, CA, United States) at 37°C with 5% CO_2_. Cells were transfected using Lipofectamine 2000 Reagent (Thermo Fisher Scientific, Waltham, MA, United States) at 80–90% confluence. A total of 1 μg of lamprey ENaC DNA per well were transfected with plasmid ratio (ENaCα: ENaCβ: ENaCγ = 1:1:1). Briefly, total mixed DNA constructs were diluted in 250 μl Opti-MEM (Thermo Fisher Scientific, Waltham, MA, United States of), and 2 μl Lipofectamine 2000 was diluted in 250 μl Opti-MEM; afterward, the diluted DNA and diluted Lipofectamine 2000 were incubated at room temperature for 5 min then mix gently. After this mixture medium was incubated at room temperature for another 20 min, the total 500 μl mixture medium was added to each well. Cells were seeded at 35–70 × 10^3^ cells/well on poly-L-lysine-coated coverslips in a 24-well plate 1 day after transfection. Cells were then applied for electrophysiological whole-cell patch recordings 1–2 days after being seeded. For control experiments, cells were transfected with 1 μg of pEYFP-C1 plasmid only.

The whole-cell patch recordings were carried out at room temperature using pipettes with 2–5 MΩ resistance. Pipettes were pulled with a P-97 micropipette puller (Sutter Instrument, Novato, CA, United States) from filamented borosilicate pipettes (G150TF-3, Sutter Instrument). The extracellular solution contained: 150 mM NaCl, 2 mM MgCl_2_, 1 mM CaCl_2_, 10 mM HEPES; pH 7.4. The intracellular solution contained: 120 mM CsCl, 5 mM NaCl, 2 mM MgCl_2_, 5 mM EGTA, 10 mM HEPES, 2 mM Mg-ATP; pH 7.4. Briefly, Cells membrane potential was held at −60 mV and stepped to test potential from −160 mV to +60 mV in increments of 20 mV. Signals were amplified using Axopatch 200B amplifier (Molecular Devices, Foster City, CA, United States), low-pass filtered at 2 kHz, digitized at 10 kHz by a Digidata 1440A converter (Molecular Devices), and acquired with software Clampex 10 (Molecular Devices).

### mRNA *in situ* hybridization and immunocytochemical staining

Previously cloned *scnn1a*, *scnn1b*, and *scnn1g* fragments in the pGEM-T vector were amplified by PCR using T7 and SP6 primers. DIG-labeled RNA sense and anti-sense probes were then synthesized by *in vitro* transcription with T7 and SP6 RNA polymerase (Roche, Penzberg, Germany). The quality and concentrations of digoxigenin (Dig)-labeled RNA probes were examined using RNA gels and a dot-blot assay.

To obtain high and significant signals of mRNA and protein, the lampreys acclimated to low-Na^+^ FW for 7 days were sampled. The gills were fixed in Bouin solution for 24 h followed by one rinse in 70% ethanol. Samples were fully dehydrated through a graded ethanol series and embedded in Paraplast (Paraplast Plus, Sigma, P3683). Sections were cut at a thickness of 7 μm on a Leica RM2245 microtome. Horizontal sections (vertical to vascular) were collected on poly-L-lysine-coated slides for further experiments or storage. The slides were deparaffinized in Histoclear II^®^ for 4 min, then passed through a descending alcohol series (100%, 95%, and 50% for 2 min each). Deparaffinized samples were initially treated with 10 µg/ml proteinase K (in PBS) at room temperature for 5 min and then refixed in 4% PFA (in PBS) at room temperature for 20 min. Samples were then washed in a descending rinse with PBST (0.1% Tween 20). Afterward, samples were washed with PBST and then incubated in hybridization buffer (HyB) containing 50% formamide, 5× SSC buffer, 0.092 M citric acid, and 0.1% Tween-20 in H_2_O with DEPC for 5 min at 65°C. Prehybridization was performed in HyB^+^ (a hybridization buffer supplemented with 500 µg/ml yeast tRNA and 5 μg/ml heparin) for 2 h at 65°C. After prehybridization, samples were hybridized with respective RNA probes diluted 1:25 in HyB^+^ at 65°C overnight. Samples were then washed with 2× SSC (at 65°C for 15 min, twice), followed by 0.2× SSC (at 65°C for 30 min, twice). After serial washings, samples were incubated for 4 h in a preblocking solution. An alkaline phosphatase (AP)-coupled anti-DIG antibody (1:500) and BM Purple (Roche Applied Science, Indianapolis, IN, United States) were used to detect the signals. *In situ* hybridizations were performed with respective sense probes as the negative control.

For immunohistochemical staining, the *in situ* hybridized samples were incubated in 5% blocking solution for 2 h to prevent non-specific binding. Samples were then incubated overnight at 4°C with Na^+^-K^+^-ATPase (NKA) α5-monoclonal antibody (anti-avian NKA α subunit diluted 1:400 with 1% blocking solution; Developmental Studies Hybridoma Bank, University of Iowa, Ames, IA). Moreover, PBST-washed (5 min, three times) slides were incubated in goat anti-mouse IgG conjugated with Alexa Fluor 488 (diluted 1:500 with PBST, Molecular Probes, Carlsbad, CA, United States). To allow double-color immunofluorescence staining with V-type H^+^-ATPase (VHA), the immunostained slides were washed (5 min, three times) in PBST and then incubated in 5% blocking solution for 2 h. Polyclonal antibody against zebrafish VHA subunit A (diluted 1:400 with 1% blocking solution; 17115-AP, proteintech, United States) was applied for overnight incubation at 4°C. Afterward, PBST-washed slides (5 min, three times) were incubated in goat anti-rabbit IgG conjugated with Alexa Fluor 568 (diluted 1:500 with PBST, Molecular Probes). Finally, images were acquired using an upright microscope (Axioplan 2 Imaging; Carl Zeiss).

To quantify the density of NHE3-expressing ionocytes, immunocytochemical staining was performed on medaka embryos according to a previous study (Hsu et al., 2014). The samples were incubated with a specific SLC9A3 (NHE3) polyclonal antibody against the C-terminus (VAPSQRAQTRPPLT AG) of medaka NHE3 protein (diluted 1:1,000) overnight at 4°C. After PBS washing, the samples were further incubated in goat anti-mouse IgG conjugated with Alexa Fluor 488 (diluted 1:500 with PBST, Molecular Probes, Carlsbad, CA, United States). The NHE3-labeled cell number on the yolk sac surface was calculated per mm^2^ for each individual.

### Scanning ion-selective electrode technique

Na^+^ and NH_4_^+^ fluxes at the ionocytes in medaka yolk skin were measured by SIET using methods described in previous studies (Wu et al., 2010; Shih et al., 2012). Briefly, probes consisted of Na^+^ and NH_4_^+^ selective microelectrodes (tip diameter, 3–5 μm) that respectively contained Na^+^ ionophore II cocktail A (100 mM NaCl) and NH_4_^+^ ionophore I cocktail B (100 mM NH_4_Cl) (Sigma-Aldrich, Saint Louis, United States). Each microelectrode was confirmed to have a Nernst slope of 56–59 before measurements were made. Electrode vibration and positioning were controlled with a stepper motor-driven three-dimensional (3D) positioner (Applicable Electronics). To record the ion flux of ionocytes, the microelectrode was moved to a position about 2 μm above the apical surface of the ionocytes in intact embryos incubated in the recording medium (0.5 mM NaCl, 0.2 mM CaSO_4_, 0.2 mM MgSO_4_, 300 μM MOPS buffer, and 0.3 mg/L ethyl 3-aminobenzoate ethanesulfonate). ASET software (Science Wares, East Falmouth, United States) was used for data acquisition, preliminary data processing, and the 3D electrode positioner control.

For inhibitor experiments, 5-ethylisopropyl amiloride (EIPA; MedChemExpress) was dissolved in dimethyl sulfoxide (DMSO, Sigma-Aldrich). Medaka embryos were incubated with 0.1% EIPA or DMSO (control) for 10 min. After a brief rinse, the embryos were immediately moved to the recording medium for SIET measurement. For the high ammonia experiments, the ion fluxes were first measured by SIET in embryos incubated in the recording medium. Then, after replacing the medium with high NH_4_^+^ (5 mM)-recording medium, the same embryos were subjected to another SIET measurement.

### Measurement of ammonia excretion in fish

Water for the ammonia (NH_3_/NH_4_^+^) excretion measurement was fully aerated, and experiments were proceeded under 28 ± 1°C. Fish were gently placed in a sealed glass chamber (150 ml) for the ammonia accumulation, and water samples were collected at different time points to determine the linear accumulation range. 15 min were then decided for ammonia accumulation measurement. An empty chamber without fish was used as a bacterial control. The 25 µl of water sample was mixed with 100 µl color reagent [21 mM sodium tetraborate, 0.063 mM sodium sulfite, 50 ml L^−1^ OPA in ethanol (40 g/L)] in a 96-well black microplate and incubated for 2 h at room temperature. NH_4_Cl solution was used as a standard (Holmes et al., 1999). The reaction was protected from light and read at the excitation/emission wavelengths of 350/420 nm on a Spectra Max M5 microplate reader (Molecular Devices, CA, United States). The ammonia excretion rate (μmol h^−1^ g^−1^) was calculated by the following equation:

Δ[ammonia]⋅V/T⋅W

whereas *∆[ammonia]* (μM) is the ammonia concentration of the unknown sample minus ammonia concentration of bacterial control; *V* is the total water volume of the glass chamber (150 ml); *T* is the accumulation period (15 min); *W* is the body weight of medaka (g)

### Statistical analysis

GraphPad Prism 7.00 (GraphPad, San Diego, CA, United States) was used for statistical analyses. Values are presented as mean ± SE. Student’s t-test was used to analyze the difference between treatments.

## Results

### Phylogenetic analysis of lamprey epithelial Na^+^ channel

Using putative amino acid sequences derived from various genomic databases (*in silico* prediction was accomplished with the ENSEMBL genome browser, ver. Ensembl Release 104; May 2021), we performed multiple sequence alignments and maximum-likelihood phylogenetic analyses of ENaC homologs (Figure 2B; Supplementary Table S2). ENaC homologs could be sub-divided into four clusters. Our analysis showed that the *scnn1d* gene, encoding the δ (delta) subunit in other vertebrates, is absent in both sea lamprey (*Petromyzon marinus*) and the stenohaline lamprey. Furthermore, the stenohaline lamprey ENaC genes were found to include three different subunits, alpha (*scnn1a*), beta (*scnn1b*), and gamma (*scnn1g*). The gene for each subunit was cloned from gill tissues and shown to cluster with its respective ortholog in sea lamprey (Figure 2B). Further electrophysiological experiments utilized co-expression of the three stenohaline lamprey ENaC homologs in HEK293 cells revealed that the gene product allows the flow of Na^+^ ions across the cell membrane (Figure 2C). Either amiloride or estrogen treatment significantly diminished the Na^+^ inwardly transport activity in the ENaC(α+β+γ)-expressing HEK293 cells (Figure 2C and Supplementary Figure S1).

### Expression and localization of ENaC in lamprey gills

**FIGURE 3:**
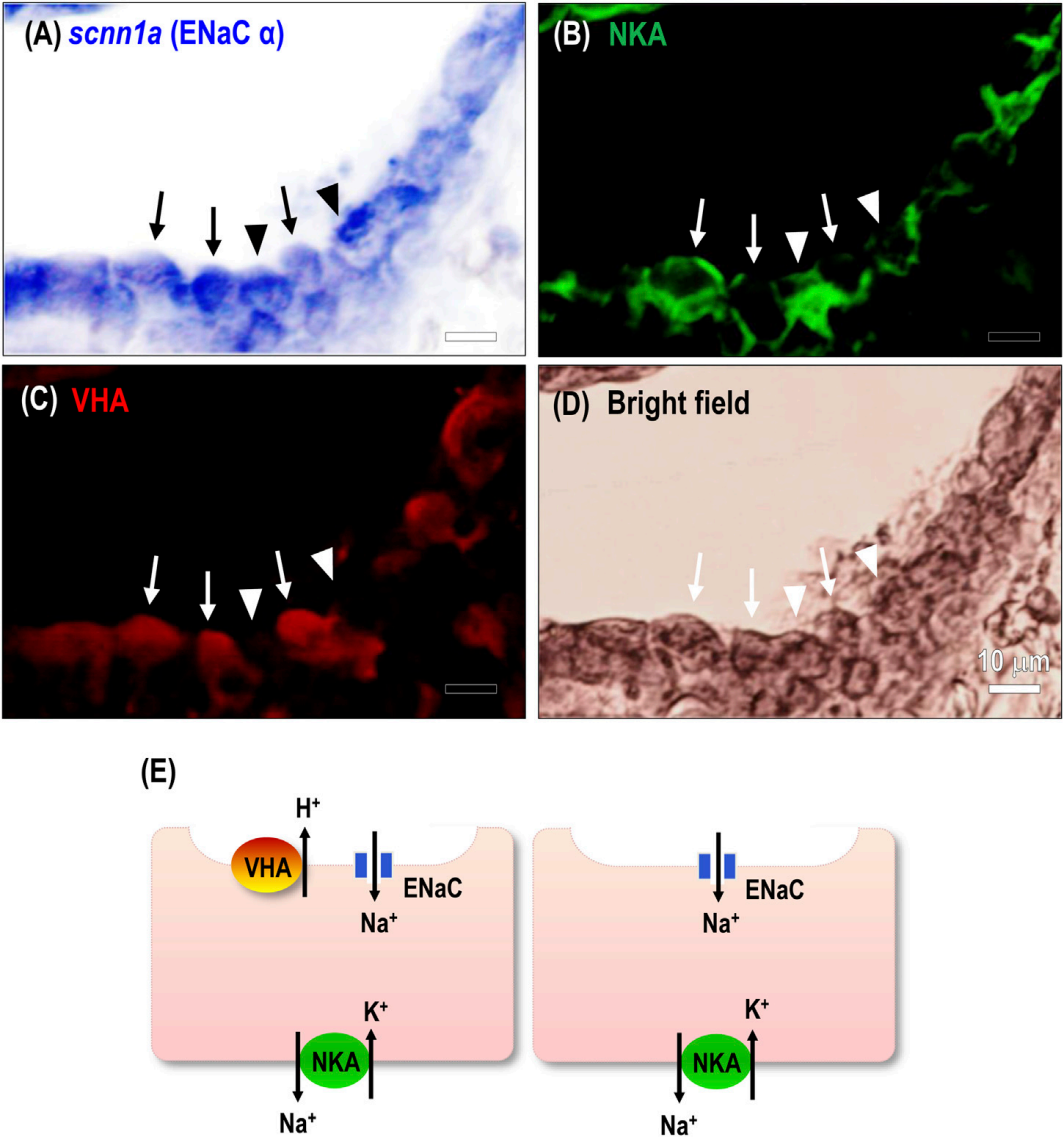
Spatial expression of ENaC in the stenohaline lamprey gills. RNA *in situ* hybridization (*scnn1a*) and immunocytochemical staining of Na^+^-K^+^-ATPase (NKA) and V-type H^+^-ATPase (VHA) was performed in gill paraffin sections. **(A–C)**, *scnn1a* mRNA (encoding ENaC alpha subunit) and NKA were co-localized in most gill epithelial cells; a great part of the cells showed VHA signals. Arrow, co-localization of the three transporters; arrowhead, cell without apical VHA signal. **(D)**, Bright-field image of gill cells. **(E)**, An illustration shows the distribution of related ion transporters in gill ionocytes.

Transcriptomic analysis was conducted on gill tissue from stenohaline lamprey acclimated to a low-Na^+^ FW environment for 7 days (Figure 2D). Transcripts of *scnn1a*/*-b*/*-g* (ENaC) and *atp6v1b/1h* (VHA) were all significantly upregulated in stenohaline lamprey under low-Na^+^ conditions compared with those in control FW, whereas expression of *slc9a3.1* (NHE3) was slightly downregulated (Figure 2D). Quantitative real-time PCR (qPCR) analysis confirmed that both ENaC and VHA mRNA levels were increased after low-Na^+^ acclimation (Figure 2E). These results suggest that ENaC and VHA may work in cooperation to promote Na^+^ uptake. Our cellular localization experiments further support the notion that ENaC and VHA act cooperatively. The mRNAs of *scnn1a*, *-b*, and *-g* were observed in most cells of gill epithelia (Figure 3A; Supplementary Figure S2). In the case of *scnn1a*, the mRNA was expressed in Na^+^-K^+^-ATPase (NKA)-labeled gill cells (Figures 3A,B). NKA is a basolateral molecular marker for certain types of ionocytes, the cells that are mainly responsible for active ion uptake or secretion in fish gills (Hwang et al., 2011). In the triple labeling experiments, only part of the gill epithelial showed apical VHA signals (Figure 3A–D), indicating that *scnn1a* is either co-expressed with apical VHA or adjacent to VHA-labeled ionocytes (Figure 3E).

### NHE3-mediated Na^+^ uptake/NH_4_^+^ excretion in medaka gills and skin

**FIGURE 4:**
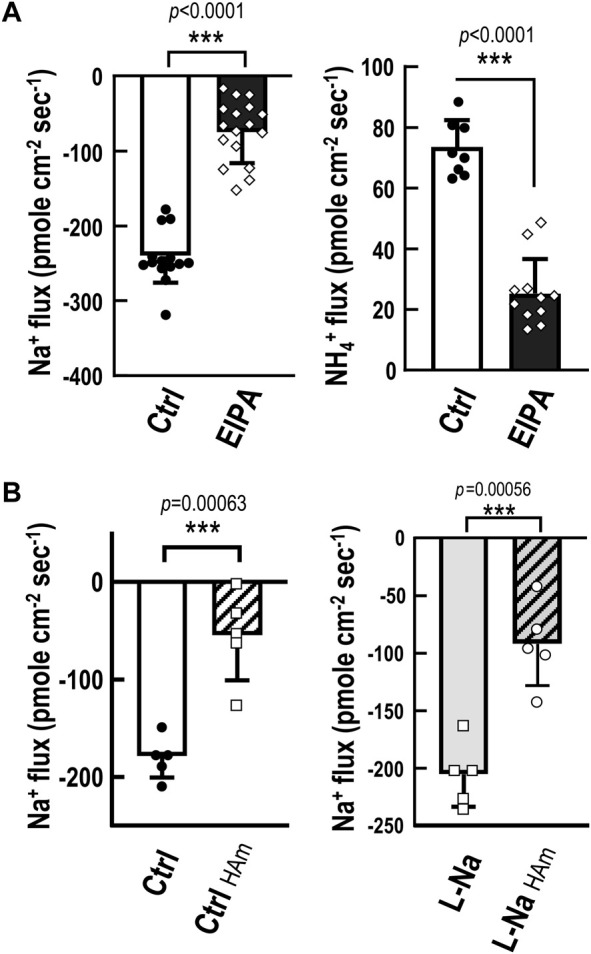
Functional characterization of NHE-mediated Na^+^ uptake/NH_4_^+^ excretion machinery. Na^+^ and NH_4_^+^ fluxes at the ionocytes in medaka yolk skin were measured by SIET. **(A)**, Treatment with 0.1% 5-ethylisopropyl amiloride (EIPA; an NHE inhibitor) for 10 min suppressed both Na^+^ influx and NH_4_^+^ efflux at the ionocytes compared with the control group (Ctrl). **(B)**, Acute exposure to 5 mM NH_4_^+^ (HAm) suppressed ionocyte Na^+^ influx in embryos acclimated to low-Na^+^ (0.01 mM, L-Na) or control freshwater (1.02 mM, Ctrl) for 7 days. Data are presented as mean ± SE. Asterisks indicate significant difference of *p* < 0.001 (***) (Student’s *t*-test).

**FIGURE 5:**
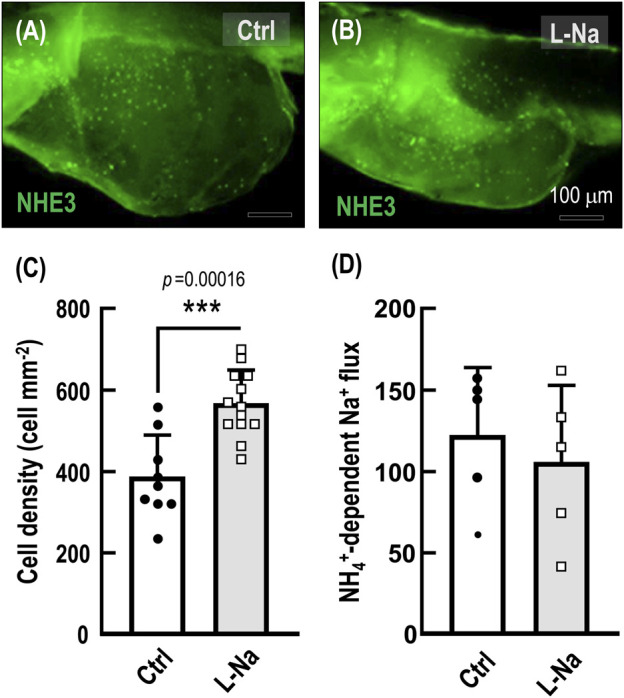
Effects of low-Na^+^ acclimation on medaka ionocyte cell density and NH_4_^+^-dependent Na^+^ uptake. Medaka embryos were acclimated to low-Na^+^ (0.01 mM, L-Na) or control freshwater (1.02 mM, Ctrl) for 7 days and then were subjected to the measurement of number of NHE3-expressing ionocytes and NH_4_^+^-dependent Na^+^ uptake at the ionocytes. **(A–C)**, Acclimation to L-Na resulted in increased cell number of Na^+^/H^+^ exchanger 3 (NHE3)-labeled ionocytes in medaka compared with that of the Ctrl group. **(D)**, The NH_4_^+^-dependent Na^+^ uptake (derived from Figure 3B) at the ionocytes did not change between the L-Na and Ctrl groups. Data are presented as mean ± SE. Asterisks indicate significant difference of *p* < 0.001 (***) (Student’s *t*-test).

The non-invasive scanning ion-selective electrode technique (SIET) to directly detect real-time ion currents of ionocytes was only established in embryonic skin, and the skin is the major site for body fluid ion regulation functions before the gill functioning in embryonic stages (Wu et al., 2010; Shih et al., 2012). Therefore, SIET was adopted to characterize the function of NHE3-mediated Na^+^ uptake and NH_4_^+^ excretion in medaka ionocytes. Na^+^ and NH_4_^+^ fluxes at the ionocytes in medaka yolk skin were measured by SIET (Figure 4). Treatment with 0.1% 5-ethylisopropyl amiloride (EIPA; an NHE inhibitor) for 10 min suppressed both Na^+^ influx and NH_4_^+^ efflux (Figure 4A) at the ionocytes compared with the control group, showing a similar inhibitory effect on both Na^+^ uptake (about 70%) and NH_4_^+^ excretion (about 65%), although the apparent Na^+^ uptake rates were higher than those of NH_4_^+^ excretion. This apparent discrepancy may have been due to the different sensitivities of Na^+^ and NH_4_^+^ probes (Wu et al., 2010). Furthermore, acute exposure to 5 mM NH_4_^+^ suppressed ionocyte Na^+^ influx in embryos acclimated to control FW (1.02 mM) or low-Na^+^ (0.01 mM) for 7 days (Figure 4B). These results suggest the role of NHE3-mediated Na^+^ uptake and NH_4_^+^ excretion in medaka body fluid Na^+^ homeostasis. The subsequent experiments reinforced this notion. Acclimation to low-Na^+^ for 7 days resulted in an increased cell number of NHE3-labeled ionocytes in medaka (Figures 5A–C) with no changes in the NH_4_^+^-dependent Na^+^ uptake (derived from Figure 4B) at the ionocytes (Figure 5D). This treatment results in an overall stimulation of Na^+^ uptake capacity. Furthermore, in adult medaka, acclimated to low-Na^+^ for 7 days stimulated gill mRNA expression of *slc9a3* compared with the control group (Supplementary Figure S3).

## Discussion

### Initial evolution of epithelial ENaC-mediated Na^+^ uptake

ENaC genes may have initially evolved in the earliest vertebrates (Wichmann and Althaus, 2020). Hagfish are one of the earliest extant vertebrate groups (cyclostomes, jawless vertebrates) and exclusively reside in SW. These species are incapable of absorbing Na^+^ from the environment because of constraints related to their classification as osmo- (and iono-)conformers and their inability to survive in FW (Robertson, 1963). Hagfish have ENaC genes, but the genes are putatively involved in acid-base regulation rather than body fluid Na^+^ homeostasis (Wichmann and Althaus, 2020), although physiological studies on hagfish ENaC are still lacking (Figure 1).

Lampreys are the superclass Cyclostomata and represent the most ancient group of vertebrates presumed to have invaded FW habitats (Halstead and Lawson, 1985; Yang et al., 2016). Considering their morphological evidence and evolutionary history on this planet (Wang et al., 2021), lampreys showed common features in all chordates originating from the ancestor for studying vertebrates’ origin and evolution. As a consequence, this primitive agnathan may be the most appropriate model for studying vertebrate organismic and functional differentiation. (Yang et al., 2016) and is likely to have been a pioneer in developing ENaC-mediated Na^+^ uptake function for body fluid homeostasis. (Figure 1). Therefore, we asked whether stenohaline lamprey (*L. reissneri*) exhibits molecular and physiological characteristics that would support this notion. The stenohaline lamprey ENaC genes include three different subunits, and electrophysiological experiments utilized co-expression of the three stenohaline lamprey ENaC homologs in HEK293 cells revealed that stenohaline lamprey ENaC could be functionally characterized with a conventional Na^+^ transport activity similar to mammalian counterparts with notable amiloride- and estrogen-sensitive properties.

Subsequent physiological and transcriptomic experiments were conducted to test the potential functional significance of ENaC in a primitive ancestor of modern vertebrates. Transcripts of *scnn1a*/*-b*/*-g* (ENaC) and *atp6v1b/1h* (VHA) were all significantly upregulated in stenohaline lamprey gills under low-Na^+^ conditions (compared with those in control FW) with downregulation of *slc9a3.1* (NHE3), suggesting that ENaC and VHA may work in cooperation to promote Na^+^ uptake. Reinforcing the molecular evidence to support the notion that ENaC and VHA act cooperatively, triple-labeling experiments demonstrated *scnn1a* to be either co-expressed with apical VHA or adjacent to VHA-labeled ionocytes. These results suggest that VHA may generate apical electrochemical gradients to drive Na^+^ transport through apical ENaC (Figure 3E) in the gills of stenohaline lamprey, similar to the mechanism of ENaC-mediated Na^+^ uptake reported in frog skin (Ehrenfeld and Klein, 1997; Wichmann and Althaus, 2020). As such, stenohaline lamprey may be one of the pioneers in developing the trait of VHA-driven ENaC Na^+^ uptake (abbreviated as “the ENaC trait” in the following text) for body fluid Na^+^ homeostasis during vertebrate evolution.

### Conservation and loss of the ENaC trait

As previously mentioned, lamprey may be one of the first vertebrate groups that initially became equipped with the ENaC trait to cope with novel FW environments, which are hypotonic and Na^+^-poor compared with the original SW habitats. In this context, it is reasonable to expect that the ENaC trait was developed in the vertebrates inhabiting FW (Figure 1). Nevertheless, ENaC genes do exist in the genome of Chondrichthyes (cartilaginous fish) (Venkatesh et al., 2007), and some euryhaline cartilaginous fish may absorb Na^+^ against an unfavorable gradient in FW (Pang et al., 1977). The gills and kidney are the major organs for ion uptake in FW elasmobranchs (Ballantyne and Robinson, 2010); however, there have been no studies on ENaC-mediated Na^+^ uptake in these organs so far (Reilly et al., 2011; Imaseki et al., 2019) (Figure 1). On the other hand, the ENaC trait has been reported in other groups of vertebrates, including lobe-finned fish and tetrapods (Figure 1). In lungfish, ENaC is expressed on the apical membrane of NKA-labeled gill cells, and the overexpression of ENaC in *Xenopus* oocytes revealed that it has a Na^+^ transport function (Uchiyama et al., 2011). Like fishes, amphibians absorb Na^+^ from FW environments through the skin in order to compensate for epithelial ion loss resulting from outward Na^+^ gradients (from the body fluid to FW) (Ehrenfeld and Klein, 1997). Na^+^ uptake is mainly achieved by ENaC-mediated machinery in skin cells (Ehrenfeld and Klein, 1997). In humans, ENaC is mainly localized to the apical membrane of principal cells in the distal part of the kidney, where it plays a vital role in fine-tuning body fluid Na^+^ homeostasis, a key physiological process in the regulation of extracellular fluid volume and blood pressure (Palmer and Schnermann, 2015). As such, the ENaC-mediated Na^+^ uptake machinery may have been utilized in various osmoregulatory organs of most vertebrates, beginning with certain evolutionary transitions (Figure 1). Nevertheless, teleosts and their sister groups, holostei and chondrostei, do not have ENaC genes, in contrast with most vertebrates (Wichmann and Althaus, 2020). Thus, these groups do not exhibit the ENaC trait for body fluid Na^+^ homeostasis (Figure 1).

### Teleosts may rely on Na^+^/NH_4_^+^ exchange for Na^+^ uptake

The reason for the loss of ENaC in some species is unclear, but it has been proposed that alternative pathways may have replaced the Na^+^ uptake function of ENaC in teleosts. Despite this plausible explanation, no convincing evidence has yet been put forth to support this notion (Venkatesh et al., 2007; Wichmann and Althaus, 2020). In teleosts, there are several transport pathways reported to be involved in Na^+^ uptake from the FW environment. Pharmacological or loss-of-function studies have proposed a trait of apical VHA-driven Na^+^ uptake machinery in several species, including zebrafish (Esaki et al., 2007), rainbow trout (Reid et al., 2003), tilapia and carp (Fenwick et al., 1999). However, convincing molecular evidence of the identity of Na^+^ uptake transporter(s) is still lacking. Moreover, only zebrafish and very limited species reveal apical VHA in gill or skin ionocytes with convincing localization evidence (Tseng et al., 2020). This VHA-driven Na^+^ uptake machinery is still an open question to be answered by molecular and cell biological approaches in the future. Acid-sensing ion channel (ASIC), another member of the degenerin/ENaC superfamily, was proposed as a possible alternative pathway for Na^+^ uptake in the gills of teleosts (Dymowska et al., 2014); however, the loss-of-function experiments failed to confirm the role of ASIC in fish Na^+^ uptake (Zimmer et al., 2018), and the functional significance of ASICs in fish gill Na^+^ uptake from FW has been questioned with respect to their characteristic gating kinetics (Wichmann and Althaus, 2020). It, therefore, precludes us from drawing a conclusive role about the function of ASICs in teleost body fluid Na^+^ homeostasis. Apical NHE3 and NCC have been well characterized to be responsible for Na^+^ uptake from the FW environment in adult gills or larval skin, and NHE3 may absorb the majority of Na^+^, with NCC playing only a minor or backup role, as was shown in FW zebrafish (Esaki et al., 2007; Chang et al., 2013). It is, therefore, reasonable to presume the NHE-mediated machinery could be a possible candidate to have replaced the use of ENaC for body fluid Na^+^ homeostasis in teleosts. In an overexpression study, teleost NHE3 was demonstrated as an ammonia transporter other than Rh proteins and to promote Na^+^ uptake from Na^+^-poor FW through Na^+^/NH_4_^+^ exchange down the NH_4_^+^ gradient (Ito et al., 2014). Of note, teleosts are ammonotelic and excrete much more ammonia than other vertebrates (Evans et al., 2005) (Table 1). Accordingly, we used Japanese medaka (*Oryzias latipes*) as a model to test the hypothesis that teleosts rely on NHE-mediated Na^+^/NH_4_^+^ exchange as the dominant pathway for Na^+^ uptake. Medaka is similar to many other teleosts in that they highly express NHE3 in a specific group of gill (or larval skin) ionocytes, which are responsible for maintaining body fluid ionic and acid-base homeostasis *via* Na^+^/H^+^ or NH_4_^+^ exchange (Hsu et al., 2014; Tseng et al., 2020). Analyses using SIET demonstrated that the NHE-mediated pathway plays a major role in Na^+^ uptake (about 70–71% of the total uptake) in medaka ionocytes (Figure 4; Table 1). This portion of Na^+^ uptake appears to be mainly achieved by Na^+^/NH_4_^+^ exchange based on several lines of evidence. First, EIPA, an NHE-specific inhibitor, showed a similar inhibitory effect on both Na^+^ uptake (about 70%) and NH_4_^+^ excretion (about 65%) (Figure 4A). Exposure to high ammonia (5 mM) is known to suppress most of the Na^+^/NH_4_^+^ exchange in fish ionocytes (Wu et al., 2010; Shih et al., 2012), and exposure of medaka to this condition caused about a 71% decline in Na^+^ uptake (Figure 4B). Together, these results suggest the NHE-mediated Na^+^/NH_4_^+^ exchange to be an important pathway for Na^+^ uptake in fish ionocytes; however, more species should be studied to support this hypothesis. Futhermore, the reliance on NHE-mediated Na^+^/NH_4_^+^ exchange for body fluid Na^+^ homeostasis uptake may vary by species, as teleosts (and other ENaC-loss groups) exhibit a wide variety of physiological capabilities (or constraints) (e.g., metabolic features, homeostatic strategies, and hormone expression characteristics) and environments that they inhabit.

**TABLE 1:** Physiological parameters related to ammonia metabolism in different vertebrates.

	Lamprey gills	Teleost gills	Amphibrian skin	Mammalian kidney (proximal tubule)
Major nitrogen waste	Ammonia	Ammonia	Ammonia/urea	Urea
NH_4_^+^ excretion rate (μmole/kg/h)	50–100^a^	300∼2,500^b^	10∼120^f^	24∼30^h^
External NH_4_^+^ concentration (μM)	FW < 0.6	FW < 0.6	FW < 0.6	Lumen 480–540^i^
Intracellular NH_4_^+^ concentration (μM)	—	626∼963^c^	—	100^j^
Plasma NH_4_^+^ concentration (μM)	—	208∼583^d^	397 ± 2^g^	80^i^
NH_4_^+^ excretion-dependent Na^+^ uptake/total Na^+^ uptake (%)	—	70∼71^e^	25^f^	0.16∼0.20^k^

Data sources.

a Data from (Wright, 2007).

b Present study and the data from (Hillaby and Randall, 1979; Mcdonald and Wood, 1981; Brix et al., 2013).

c Data from (Ogata and Murai, 1988; Wright et al., 1988).

d Data from (Hillaby and Randall, 1979; Ogata and Murai, 1988; Wright et al., 1988).

e Data estimated in the present study.

f Data from (Romeu and Salibián, 1968).

g Data from (Balinsky and Baldwin, 1961).

h Estimation from the data of (Hamm et al., 2015).

i Data from (Good and Dubose, 1987).

j Data from rat erythrocyte (Albano and Francavilla, 1971).

k Estimation from the data of (Boron and Boulpaep, 2002; Hamm et al., 2015; Palmer and Schnermann, 2015).

**—**Data unavailable.

### Possible advantages of using Na^+^/NH_4_^+^ exchange to absorb Na^+^

As suggested above, NHE-mediated Na^+^/NH_4_^+^ exchange may be an alternative pathway to replace the lost ENaC in teleosts. The next question is what the possible advantages for teleosts to replace ENaC with the trait of NHE are. Accordingly, we made comparisons of major Na^+^ uptake mechanisms among different vertebrates (Table 1) to test a hypothesis that the trait of NHE-mediated Na^+^/NH_4_^+^ exchange is more efficient or energy-saving machinery than that of ENaC to absorb Na^+^ from the FW environment. In human collecting duct cells, ENaC transports Na^+^ across the apical membrane down electric and Na^+^ gradients (lumen, 40 mM; intracellular, 10 mM) (Boron and Boulpaep, 2002) that are created by basolateral NKA. For aquatic vertebrates inhabiting FW, such as lampreys, frogs and teleosts, the Na^+^ concentration (<1 mM) in the FW environment is much lower than the intracellular concentration (6 mM) (Ehrenfeld and Klein, 1997). This difference results in an outward Na^+^ gradient that is unfavorable for Na^+^ uptake through apical Na^+^ transporters. This unfavorable Na^+^ gradient appears to be one of the major constraints on the evolution of Na^+^ uptake mechanisms in vertebrates inhabiting the FW environment. As a pioneer invading FW habitats, lamprey developed an energy-consuming trait of VHA-driven ENaC Na^+^ uptake to overcome the unfavorable Na^+^ gradient (Figure 3E). A similar evolutionary event occurred in amphibians. In frog skin cells, VHA was found to hyperpolarize the apical membrane and facilitate ENaC-mediated Na^+^ transport against the unfavorable outward Na^+^ gradient (Ehrenfeld and Klein, 1997) (Figure 1). Additionally, VHA consumes ATP to hyperpolarize the apical membrane and drive Na^+^ influx through ENaC in the gills or skin of aquatic vertebrates, facilitating their conquering of low-Na^+^ habitats. Notably, the trait of VHA-driven ENaC activity in aquatic vertebrates appears to be more energy-consuming than the strategy adopted by terrestrial mammals, i.e., ENaC transport of Na^+^ down a favorable inward Na^+^ gradient. Taking all into consideration, one possible reason for the loss of ENaC in teleosts may be that, energetic efficiency was improved by selecting newly evolved energy-saving types of machinery, such as NHE-mediated Na^+^/NH_4_^+^ exchange. This slective traits appears to be energetically advantageous for teleosts to maintain body fluid Na^+^ homeostasis during their evolution in FW environments.

**FIGURE 6:**
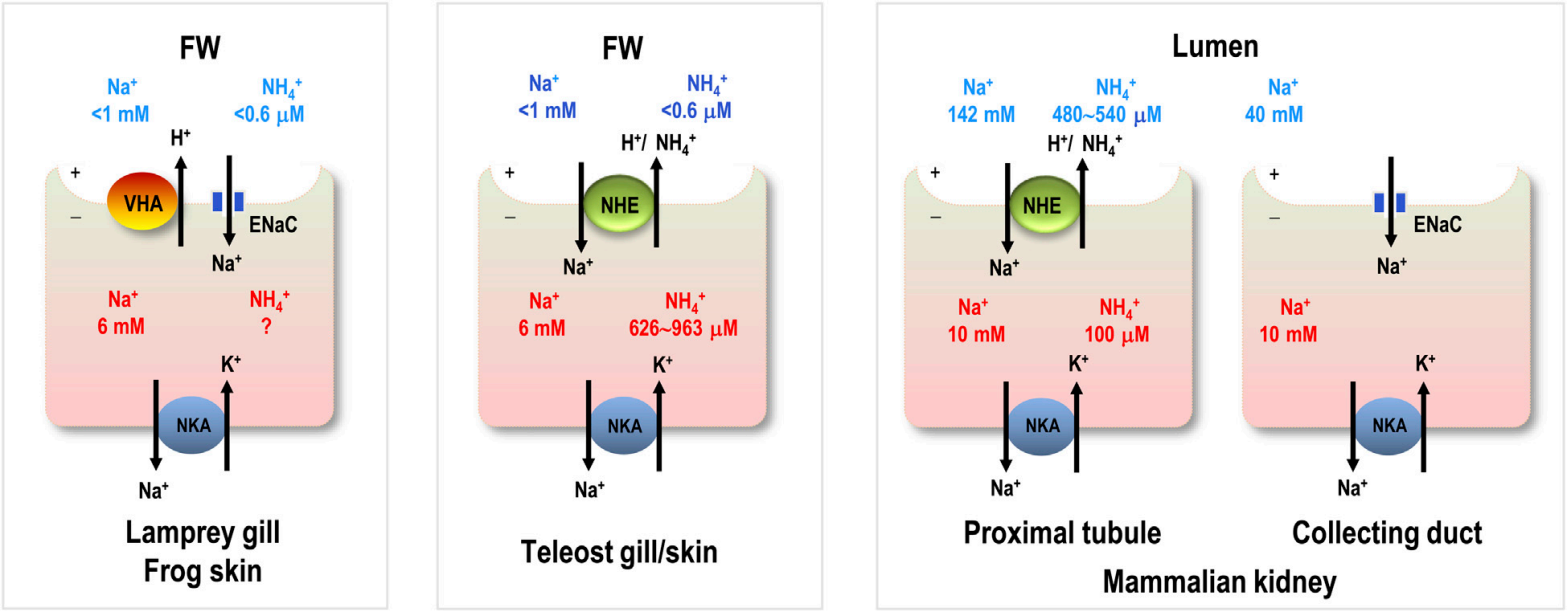
Comparison of major Na^+^ uptake machinery among different. vertebrates. Na^+^ and NH_4_^+^ concentrations in freshwater (FW) habitats are from the local natural habitats. Intracellular Na^+^ in aquatic vertebrate cells is from frog skin. Intracellular NH_4_^+^ in teleost gill is from trout blood cells. Extracellular (lumen) and intracellular Na^+^ and NH_4_^+^ concentrations in humans are from the kidney and blood cells. Intracellular NH_4_^+^ in lamprey gill and frog skin is unknown (?). ENaC, epithelial Na^+^ channel; VHA, V type H^+^-ATPase; NHE, Na^+^/H^+^ exchanger; NKA, Na^+^-K^+^-ATPase.

While Na^+^/NH_4_^+^ exchange by NHE has also been developed in vertebrates that use the ENaC as a pathway for body fluid Na^+^ homeostasis (such as lampreys, other non-teleost fishes, frogs and humans), the ENaC-adopting vertebrates may also rely on Na^+^/NH_4_^+^ exchange for a lesser (or minor) part of their total Na^+^ uptake (Figure 6). Humans are urotelic vertebrates and generally absorb about 25,400 mmol/d of Na^+^ [estimated from (Boron and Boulpaep, 2002; Palmer and Schnermann, 2015)] with only about 40–50 mmol/d of NH_4_^+^ excretion in the proximal tubule (estimated for a 70 kg adult from (Hamm et al., 2015)), indicating that Na^+^/NH_4_^+^ exchange machinery plays an exceedingly minor role (0.16–0.20%) in renal Na^+^ uptake (Table 1; Figure 6). In amphibians, skin NH_4_^+^ excretion rates are correlated with the transition from ammonotelism (more aquatic inhabitation) to urotelism (more terrestrial inhabitation) (Romeu and Salibián, 1968). Amphibians excrete NH_4_^+^ at rates of 10–120 μmol/kg/h through their skin, which is between the rates of excretion through human kidneys and teleost gills (Table 1; Figure 6). In the skin of American frog (*Leptodactylus ocellatus*), only 25% of Na^+^ uptake is derived from Na^+^/NH_4_^+^ exchange (Romeu and Salibián, 1968), while in the Japanese black salamander (*Hynobius nigrescens*), NHE3 is expressed in the kidney but not in the external gills and skin (Kumano et al., 2008; Uchiyama et al., 2011). These observations indicate that NHE-mediated Na^+^/NH_4_^+^ exchange plays a minor role in the Na^+^ uptake of amphibian skin and external gills (Table 1). Lamprey is an ammonotelic animal (Wright, 2007), and the rate at which it excretes NH_4_^+^ is between those of amphibians and teleosts (Table 1). Similar to amphibian skin and external gills, lamprey gills express a very low level of NHE3 (compared with teleosts) (Tseng et al., 2020), suggesting a minor role of NHE3 in gill Na^+^ uptake, although the exact ratio of the Na^+^/NH_4_^+^ exchange-mediated Na^+^ transport to total Na^+^ uptake in lamprey gills is unclear (Table 1). By contrast, Japanese medaka, a euryhaline teleost, utilizes NHE-mediated Na^+^/NH_4_^+^ exchange in gill and skin ionocytes as a dominant pathway of Na^+^ absorption for body fluid homeostasis (Table 1). As teleosts have lost ENaC but are capable of NH_4_^+^ excretion, they may have evolved a greater reliance on NHE-mediated Na^+^/NH_4_^+^ exchange for maintenance of body fluid Na^+^ homeostasis during their evolution in the FW system. As such, teleosts appear to take advantage of their ammonotelic significance (synthesizing plentiful ammonia as the major nitrogenous waste product) to create a favorable NH_4_^+^ gradient for driving NHE-mediated Na^+^ uptake machinery. Thus, the utilization of NHE trait for body fluid Na^+^ homeostasis is likely to be a benefit strategy for energy saving strategy and efficient absorption of Na^+^ from the FW habitats against an unfavorable Na^+^ gradient.

Therefore, the selection of NHE-mediated Na^+^/NH_4_^+^ exchange for Na^+^ uptake with simultaneous loss of ENaC and VHA-driven ENaC machinery could be an energy constraint for evolved environmental evolution selection. Indeed, gene loss has been reported as another source of genetic variation that gives rise to adaptive phenotypic diversity (Albalat and Cañestro, 2016), and selection was suggested to be a significant driver of gene loss in bacteria (Koskiniemi et al., 2012). Additionally, the availability of alternative pathways has been reported to facilitate gene loss in many organisms, including mammals and other vertebrates (Albalat and Cañestro, 2016). As the largest and most diverse vertebrate subgroup, teleosts account for half of all extant modern vertebrates (Ravi and Venkatesh, 2018). This energy constraint-driven adaptive evolution in teleosts suggests that the physiological and molecular features of NHE trait utilization were likely to compensate for the loss of the ENaC trait for maintaining body fluid homeostasis and environmental adaptation.

## Conclusion and perspectives

Evolutionary changes may be interpreted as the consequences of ecological scenarios involving stressful environments (Parsons, 2005). Since energy supply is one of the major constraints on physiological processes, trade-offs between energy allocations to different physiological processes under the energy efficiency concern can have major consequences on the ability of a population to survive and reproduce in a stressful world, such as individual fitness (Parsons, 2005; Sokolova, 2013). Selection for energy efficiency to maximize fitness in a stressful environment is a key component of evolutionary organism-environment interactions (Parsons, 2005). The invasion and colonization of FW habitats, which are much harsher than the original SW in terms of ionic strength and pH, was one of the most important advances in the evolution of vertebrate physiology (Halstead and Lawson, 1985). The evolutionary changes from the trait of ENaC to NHE-mediated Na^+^/NH_4_^+^ exchange for internal Na^+^ homeostasis and from HA-mediated H^+^ secretion to NHE-mediated NH_4_^+^ excretion for acid-base regulation (Tseng et al., 2020) exemplify the selection of energy-efficient physiological traits in teleosts to accommodate for Na^+^-poor environmental stress. Teleosts are the largest and most diverse group of vertebrates, and their evolutionary success has long been ascribed to a TWGD; however, the direct involvement of TWGD remains un-demonstrated in an astonishing array of morphological, physiological, and behavioral adaptations (Glasauer et al., 2014; Ravi and Venkatesh, 2018). Although studies on more species are needed, our findings may provide new physiological insights into this issue. Teleosts appear to leverage the advantages of ammonotelism by synthesizing a large amount of ammonia as the driving force to absorb Na^+^ and secrete acid against unfavorable ion gradients in FW habitats. These physiological traits changes lead to better energy efficiency could have contributed to the evolutionary success of teleosts since the innovations may have enabled teleosts to allocate more energy to growth, reproduction and various physiological processes, rather than ionic/acid-base regulation in FW habitats.

## Data Availability

The datasets presented in this study can be found in online repositories. The names of the repository/repositories and accession number(s) can be found below: https://www.ncbi.nlm.nih.gov/, PRJNA794988.
