# Supplementary material for: Teleostean fishes may have developed an efficient Na+ uptake for adaptation to the freshwater system
Source: Front Physiol. 2022 Oct 5;13:947958. doi: 10.3389/fphys.2022.947958 (PMC9581171; doi:10.3389/fphys.2022.947958)
Supplement: Supplementary file 1 [file Table1.DOCX]

**Supplemental Table S1** Ionic compositions (mM) in the artificial freshwater (FW)

| **Medium** | **[Na^+^]** | **[Cl^-^]** | **[K^+^]** | **[Ca^2+^]** | **[Mg^2+^]** | **[SO_4_^2-^]** |
| --- | --- | --- | --- | --- | --- | --- |
| Control FW | 1.02 ± 0.01 | 1.03± 0.00 | 0.21± 0.01 | 0.70± 0.01 | 0.31± 0.00 | 1.03± 0.01 |
| Low-Na^+^ FW | 0.01± 0.00 | 1.02± 0.00 | 0.21± 0.01 | 0.72± 0.02 | 0.31± 0.01 | 0.58± 0.02 |

Mean ± SD (*N* = 4) is indicated.
