# Supplementary figures and images for: Teleostean fishes may have developed an efficient Na+ uptake for adaptation to the freshwater system
Source: Front Physiol. 2022 Oct 5;13:947958. doi: 10.3389/fphys.2022.947958 (PMC9581171; doi:10.3389/fphys.2022.947958)

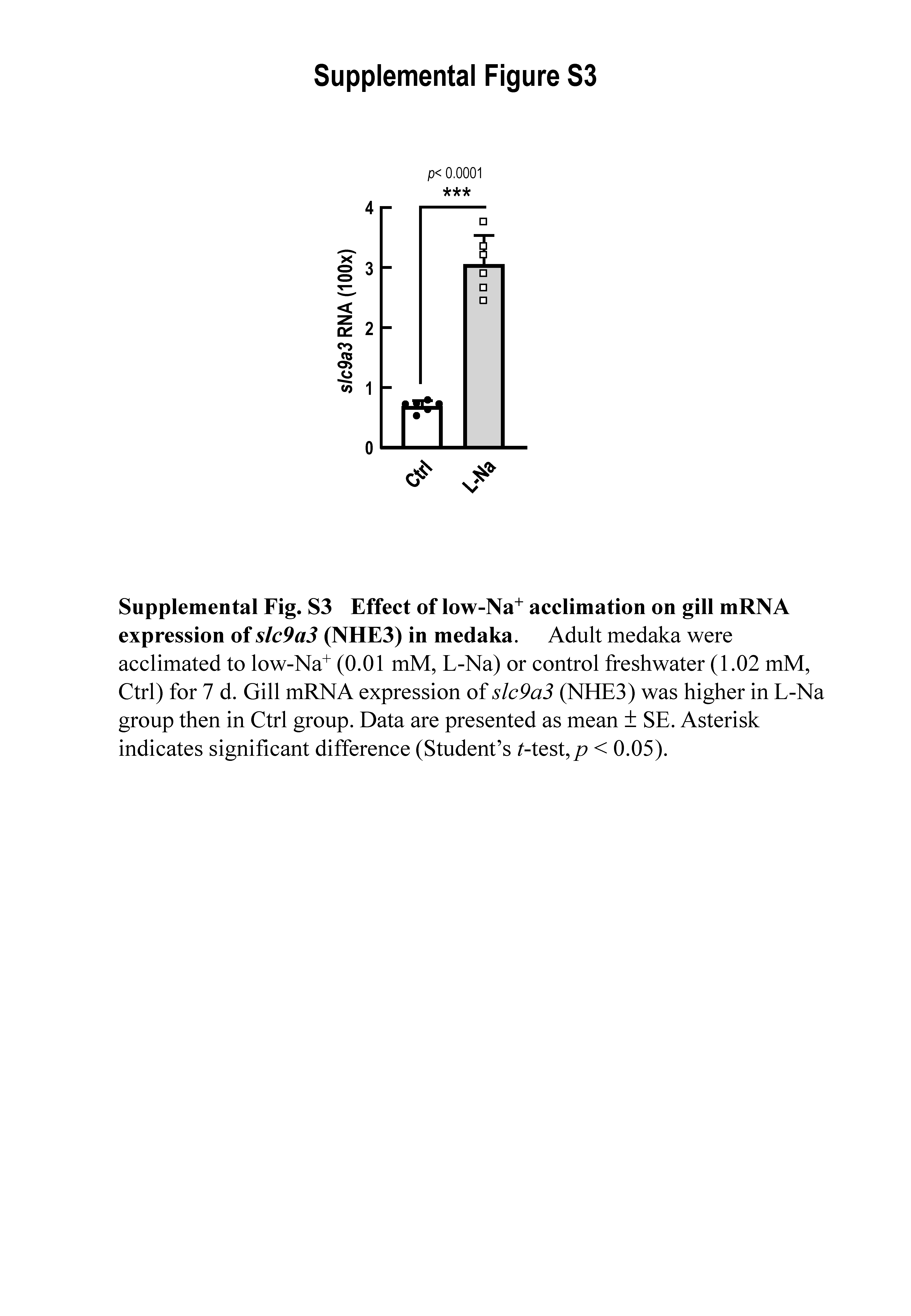

Supplement: Supplementary file 2 [file Image3.TIF]

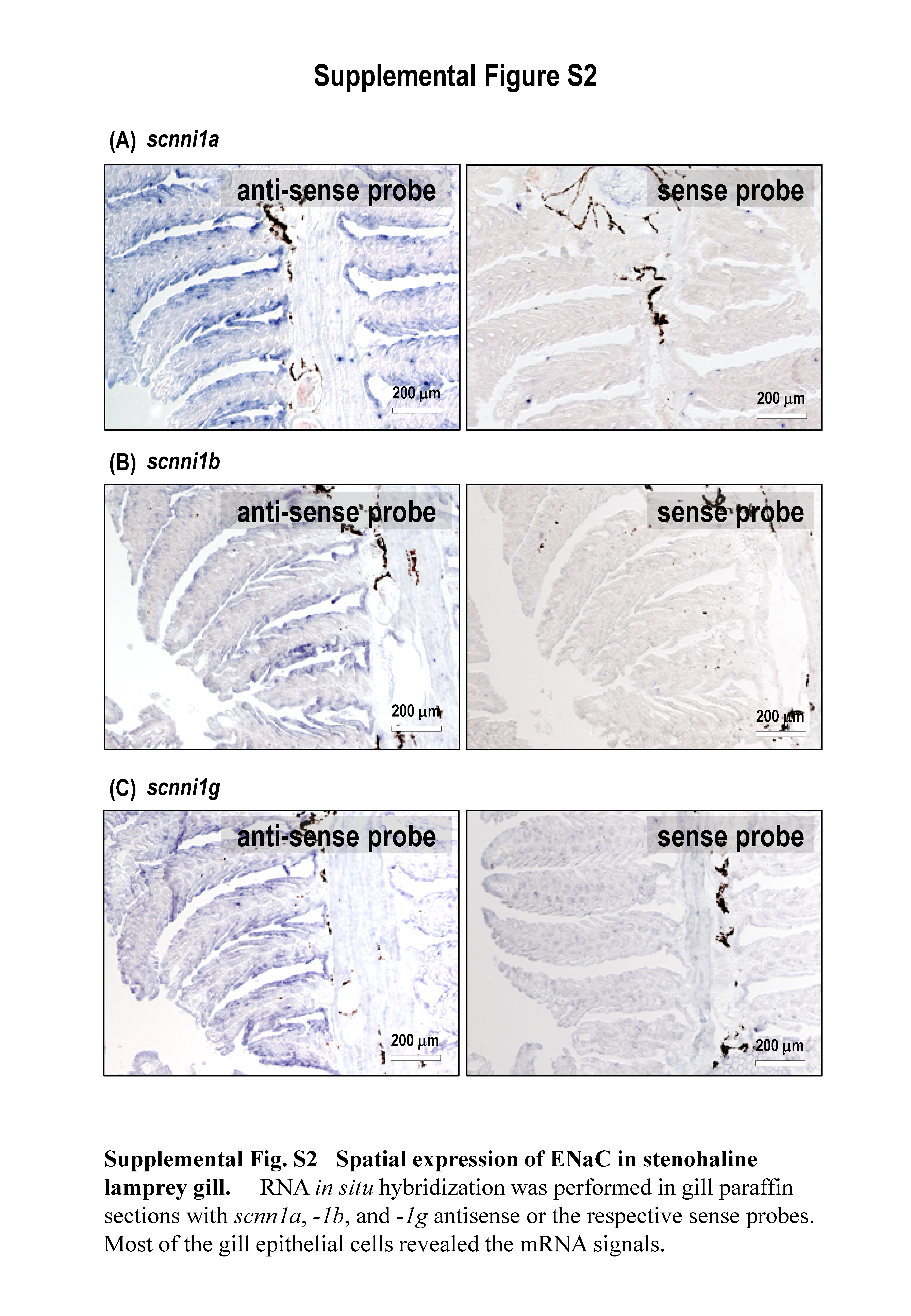

Supplement: Supplementary file 3 [file Image2.TIF]
