# Supplementary material for: Teleostean fishes may have developed an efficient Na+ uptake for adaptation to the freshwater system
Source: Front Physiol. 2022 Oct 5;13:947958. doi: 10.3389/fphys.2022.947958 (PMC9581171; doi:10.3389/fphys.2022.947958)
Supplement: Supplementary file 5 [file Table2.DOCX]

**Supplemental Table S2** Known and predicted homologs of ENaC protein family

| **ENaCα (SCNN1a) orthologs** |  |  |  |
| --- | --- | --- | --- |
| **Gene name** | **Species** | **Gene locus** | **Accession number** |
| Human SCNN1a | *Homo sapiens* | Ch. 12: 6.35 Mb | ENST00000360168.7 |
| Mouse SCNN1a | *Mus musculus* | Ch. 6: 127.19 Mb | ENSMUST00000176655.7 |
| Chicken SCNN1a | *Gallus gallus* | Ch. 1: 77.57 Mb | ENSGALT00000067040.2 |
| Lizzard SCNN1a | *Podarcis muralis* | Primary_assembly 17: 4.15 Mb | ENSPMRT00000030620.1 |
| Xenopus SCNN1a | *Xenopus tropicalis* | Primary_assembly 7: 1.84 Mb | ENSXETT00000060245.2 |
| Coelacanth SCNN1a | *Latimeria chalumnae* | Scaffold JH128489.1: 348.99 Kb | ENSLACT00000009737.1 |
| Sea lamprey SCNN1a | *Petromyzon marinus* | Scaffold GL48299: 2.87 Kb | ENSPMAT00000008481.1 |
| **Stenohaline Lamprey SCNN1a** | ***Lethenteron reissneri*** | **Unkown** | **MZ855253** |
| **ENaCβ (SCNN1b) orthologs** |  |  |  |
| **Gene name** | **Species** | **Gene locus** | **Accession number** |
| Human SCNN1b | *Homo sapiens* | Ch. 16: 23.28 Mb | ENST00000343070.7 |
| Mouse SCNN1b | *Mus musculus* | Ch. 7: 121.46 Mb | ENSMUST00000033161.7 |
| Chicken SCNN1b | *Gallus gallus* | Ch. 14: 7.36 Mb | ENSGALT00000010010.6 |
| Lizzard SCNN1b | *Podarcis muralis* | Primary_assembly 14: 29.67 Mb | ENSPMRT00000038360.1 |
| Xenopus SCNN1b | *Xenopus tropicalis* | Primary_assembly 9: 16.79 Mb | ENSXETT00000027176.4 |
| Elephant shark SCNN1b | *Callorhinchus milii* | Primary_assembly KI636001.1: 1.38 Mb | ENSCMIT00000030778.1 |
| Coelacanth SCNN1b | *Latimeria chalumnae* | Scaffold JH127449.1: 678.97 Kb | ENSLACT00000013870.1 |
| Hagfish SCNN1b | *Eptatretus burgeri* | Contig FYBX02010564.1: 691.94 Kb | ENSEBUT00000012291.1 |
| Sea lamprey SCNN1b | *Petromyzon marinus* | Scaffold GL481027: 2.90 Kb | ENSPMAT00000010217.1 |
| **Stenohaline Lamprey SCNN1b** | ***Lethenteron reissneri*** | **Unkown** | **MZ848193** |
| **ENaCγ (SCNN1g) orthologs** |  |  |  |
| **Gene name** | **Species** | **Gene locus** | **Accession number** |
| Human SCNN1g | *Homo sapiens* | Ch. 16: 23.18 Mb | ENST00000300061.3 |
| Mouse SCNN1g | *Mus musculus* | Ch. 17: 121.33 Mb | ENSMUST00000000221.6 |
| Chicken SCNN1g | *Gallus gallus* | Ch. 14: 7.38 Mb | ENSGALT00000047552.2 |
| Tiger Snake SCNN1g | *Notechis scutatus* | Primary_assembly ULFQ01000396.1: 131.36 Kb | ENSNSUT00000028769.1 |
| Xenopus SCNN1g | *Xenopus tropicalis* | Primary_assembly 9: 16.87 Mb | ENSXETT00000076215.1 |
| Elephant shark SCNN1g | *Callorhinchus milii* | Primary_assembly KI636001.1: 1.39 Mb | ENSCMIT00000030832.1 |
| Coelacanth SCNN1g | *Latimeria chalumnae* | Scaffold JH127449.1: 621.64 Kb | ENSLACT00000013312.1 |
| Hagfish SCNN1g | *Eptatretus burgeri* | Contig FYBX02010564.1: 670.54 Kb | ENSEBUT00000027585.1 |
| Sea lamprey SCNN1b | *Petromyzon marinus* | Scaffold GL479797: 4.94 Kb | ENSPMAT00000005615.1 |
| **Stenohaline Lamprey SCNN1b** | ***Lethenteron reissneri*** | **Unkown** | **MZ848194** |
| **ENaCδ (SCNN1d) orthologs** |  |  |  |
| **Gene name** | **Species** | **Gene locus** | **Accession number** |
| Human SCNN1d | *Homo sapiens* | Ch. 1: 1.28 Mb | ENST00000379116.10 |
| Guinea Pig SCNN1d | *Cavia porcellus* | Scaffold DS563041.1: 1.07 Mb | ENSCPOT00000011802.3 |
| Chicken SCNN1d | *Gallus gallus* | Ch. 14: 7.36 Mb | ENSGALT00000010010.6 |
| Tiger Snake SCNN1d | *Notechis scutatus* | Primary_assembly ULFQ01000158.1: 938.97 Kb | ENSNSUT00000001976.1 |
| Coelacanth SCNN1d | *Latimeria chalumnae* | Scaffold JH126562.1: 4.28 Mb Kb | ENSLACT00000021468.1 |
| Xenopus SCNN1d.1 | *Xenopus tropicalis* | Primary_assembly 7: 69.64 Mb | ENSXETT00000096717.1 |
| Xenopus SCNN1d.2 | *Xenopus tropicalis* | Primary_assembly 7: 69.67 Mb | ENSXETT00000002264.4 |
| Hagfish SCNN1g | *Eptatretus burgeri* | Contig FYBX02010221.1: 1.03 Mb | ENSEBUT00000015743.1 |
