# Supplementary material for: Teleostean fishes may have developed an efficient Na+ uptake for adaptation to the freshwater system
Source: Front Physiol. 2022 Oct 5;13:947958. doi: 10.3389/fphys.2022.947958 (PMC9581171; doi:10.3389/fphys.2022.947958)
Supplement: Supplementary file 6 [file Table3.DOCX]

**Supplemental Table S3** Primers used for RT-PCR (cloning) and quantitative real-time PCR (qPCR)

| **Protein name** | **Gene name** | **Purpose** | **Primer sequence (5'->3')** | | **Amplicon size (bp)** | **Primer efficiency (%)** |
| --- | --- | --- | --- | --- | --- | --- |
| Epithelial Na+ channel α  (ENaCα) | *scnn1a* | Cloning | F | GGTACACCTTCCAGTACGTCAA | 799 |  |
|  |  |  | R | CTCACAGTGGTGACGGAGTAAT |  |  |
|  |  | qPCR | F | CTACATCAAGCAGTCCGACG | 91 | 100-102 |
|  |  |  | R | GGCTGTATTCACTGTCGCTG |  |  |
| Epithelial Na+ channel β  (ENaCβ) | *scnn1b* | Cloning | F | GTCGTGTACGGCAAGAAGAA | 1149 |  |
|  |  |  | R | GGGCAGAGGGTACAGGTAATA |  |  |
|  |  | qPCR | F | TCCCATTCTTGACCACAATCG | 126 | 97-98 |
|  |  |  | R | CCACTTCAATCCCCACAGAG |  |  |
| Epithelial Na+ channel γ  (ENaCγ) | *scnn1g* | Cloning | F | GCTGGACTTCTACCTCAACAA | 1337 |  |
|  |  |  | R | GCAACAGCCACCCCTTAGATG |  |  |
|  |  | qPCR | F | CAGGGCATCCAACTCATACAG | 124 | 99-100 |
|  |  |  | R | CCACTTCAATCCCCACAGAG |  |  |
| V-type H^+^-ATPase  (VHA) | *atp6v1b* | qPCR | F | AAGGCAAGGACACGATGGACTACA | 158 | 100-102 |
|  |  |  | R | ATAGTCGTTGGCCAGGTTGAGGAA |  |  |
| Ribosomal protein L18  (RPL18) | *rpl18* | qPCR | F | GCCACATTCCCTGGTCCTACAAGA | 102 | 99-100 |
|  |  |  | R | TTACAAAGATGCGTCGCGGTTCAC |  |  |

F, forward primer; R, reverse primer
